# Cross-Platform Evaluation of Established NGS-Based Metabarcoding Methods for Detecting Food Fraud in Pistachio Products

**DOI:** 10.3390/foods15010124

**Published:** 2026-01-01

**Authors:** Sina Rammouz, Jochen Riehle, Ansgar Ferner, Markus Fischer, Christian Schäfers

**Affiliations:** 1 Institute for Hygiene and Environment, Marckmannstraße 129 b, 20539 Hamburg, Germany; sina.rammouz@hu.hamburg.de (S.R.); jochen.riehle@hu.hamburg.de (J.R.); ansgar.ferner@hu.hamburg.de (A.F.); 2 Hamburg School of Food Science, Institute of Food Chemistry, University of Hamburg, Grindelallee 117, 20146 Hamburg, Germany; markus.fischer@uni-hamburg.de

**Keywords:** oxford nanopore technologies, illumina, metabarcoding, pistachio, method comparison

## Abstract

Next Generation Sequencing is a constantly evolving technology whose applicability is increasingly expanding into the field of routine food analysis. In this context, metabarcoding has proven to be a powerful tool for detecting food fraud due to its ability to taxonomically classify even highly fragmented DNA from processed products. While Illumina sequencing platforms, representing second-generation sequencing technologies, are widely used for such applications, fourth-generation sequencing devices such as Oxford Nanopore Technologies’ MinION offer advantages in terms of flexibility, scalability, and simplified handling. In this study, we evaluate the transferability of an established Illumina-based metabarcoding method for the detection of pistachio adulteration in processed foods to the MinION platform of Oxford Nanopore Technology. In more detail, we transferred the established method from Illumina on both MinION and Flongle flow cells to assess sequencing accuracy, quantification potential and practical aspects such as cost-efficiency and workflow. Our results highlight the applicability of the MinION sequencing platform as a reliable and cost-effective alternative to Illumina protocols for routine food authenticity testing, enabling faster processing and broader accessibility without significantly compromising accuracy.

## 1. Introduction

Next Generation Sequencing (NGS) has been on the rise for some time now, not only in terms of development but also in terms of applicability. Thanks to continuous development, NGS has already reached its fourth generation. The various manufacturers are constantly working on improving the accuracy of current sequencing platforms and simplify their use in the laboratory. As the technology becomes less expensive, NGS has also been applied increasingly in the routine analysis of food products. Metabarcoding, for example, is a simple method for the detection of food fraud.

The term “Next Generation Sequencing” sums up all sequencing technologies that have been developed after Sanger sequencing, representing the “first-generation” sequencing technology. The Illumina technology belongs to the “second-generation” sequencing. The read lengths are limited (300 to 600 bp) but vast quantities of accurate sequencing data can be generated [1]. Larger DNA sections, e.g., whole genome sequences, must be assembled bioinformatically afterwards, but smaller barcoding regions can be completely covered with the read length of 300 bp. The sequencing technology is based on cluster amplification, sequencing-by-synthesis and image analysis on proprietary instruments [2]. Post-sequencing data processing can be performed with Illumina software or custom designed pipelines. The Single-Molecule Real-Time Sequencing (SMRT) of Pacific Biosciences (PacBio), a “third-generation” sequencing technology, is related to the sequencing-by-synthesis technology of Illumina. The PCR amplification is omitted and sequencing is performed at long single molecules [3]. One sequencing platform of the “fourth-generation” is represented by Oxford Nanopore Technologies (ONT) [4]. Here, sequencing flow cells consist of protein nanopores which are stabilized in an electrically resistant polymer membrane. Nucleotide detection relies on applying a voltage across the membrane, which contains sensors that can monitor real-time changes in ionic current as nucleotides pass through the pore while the DNA molecule moves through it [5,6,7,8]. There are different types of flow cells and chemicals, ranging from scalable sequencers (Flongle and MinION flow cell) to the highest throughput PromethION flow cells. The flow cells have different numbers of nanopores, resulting in different yields of output data. The signal from molecules passing through the nanopore structure before base determination is called a “squiggle”. Moreover, different nanopores contain different ‘readers’. At the outdated R9 nanopores the reader has been located in the middle of the barrel. The current R10 Nanopores have two readers spaced along their length. Therefore, bases within a DNA or RNA strand can contribute to the squiggle at any time. This leads to improvements in capturing signals around homopolymer regions [9]. ONT technology is capable of reading long sequences and thus the devices were not initially designed to be a major sequencing platform for short-read sequencing. Nevertheless, it is also applicable for short-read sequencing like metabarcoding [10]. Since Illumina technology has been around for quite some time, there are also far more publications on research in the field of metabarcoding with Illumina devices [11].

Metabarcoding uses short DNA fragments for taxonomic classification. These barcode regions are highly conserved during evolution but still have an adequate DNA sequence variation to allow taxonomic classification [11]. Different regions are particularly suitable for different groups of organisms. For example, the rbcL, ITS2, and matK regions are often used for plants and the 16S, COI, and cyt b regions are highly representable for microorganisms and animals [12]. First the regions are amplified during an amplicon PCR with the corresponding primers. The individual samples are then labeled with Native Barcodes (Oxford Nanopore Technologies) or indices (Illumina). This allows massively parallel sequencing. Afterwards the raw data needs to be bioinformatically processed ending up in a taxonomic assignment of sequences of the individual samples to the respective organism. One characteristic feature of the targeted barcode regions is their very short length (100–300 bp) [13], by which the metabarcoding method is particularly suitable for processed products such as highly processed foods, as the DNA is usually already fragmented. In addition, the short length allows a huge number of samples to be sequenced simultaneously and with high coverage. Moreover, the molecular identification method does not require taxonomic expertise and is relatively fast, cost-effective, and accurate [14]. Both sequencing platforms, Illumina and Oxford Nanopore Technologies, are not only used in the medical field but are also increasingly being used in food monitoring. This is mainly due to the fact that next to the fraudulent alteration and non-declaration of food ingredients, food safety is a very serious issue in food monitoring. The wrong declaration of food ingredients can be an economic issue for consumers but can also have health consequences or violate ethnic and religious guidelines. In particular, the missing declaration of allergenic substances can have life-threatening consequences for people with allergies. Ingredients are often replaced to maximize profits or due to a lack of resources, for example, due to poor harvests. High-value nuts such as pistachios are frequently subject to food fraud [15]. In processed products such as pastries (e.g., baklava, a traditional Turkish dessert), chocolate, and ice cream, a high pistachio content is often regarded as an indicator of superior quality and is reflected in the corresponding price [16]. Since pistachios are commonly ground into indistinguishable forms, they are particularly susceptible to fraudulent adulteration.

The seeds and nuts of *Juglans* spp., *Prunus* spp., *Helianthus* spp., *Pisum* spp., *Corylus* spp., and *Cucurbita* spp. are suspected of being used particularly often to adulterate pistachios, according to public surveillance and pre-screening. Many molecular biology studies dealing with pistachios focus only on short allergen-related genomic regions, which limits their ability to capture broader genetic diversity [17,18,19]. In contrast NGS provides a substantially more comprehensive data output. Its capacity to detect a wide spectrum of genomic variation, including single nucleotide variants, insertions, and deletions, and goes far beyond what conventional PCR-based assays can achieve [20]. In addition, the scalability of NGS workflows makes them advantageous for high-throughput applications, where processing many samples in parallel can be more cost-efficient than traditional PCR approaches. Moreover, existing studies often restrict the scope of adulteration to binary mixtures, typically assessing pistachio adulteration with a single additional species [21,22,23]. However, the metabarcoding approach allows the simultaneous identification of multiple taxa, in this case, various plant species. This offers a much more comprehensive view of product authenticity [24,25].

When comparing second- and fourth-generation sequencing technologies, the focus is often on evaluating sequencing accuracy and depth. This assessment is typically based on well-known gene segments from defined samples, such as the 16S gene region of microorganisms [1]. The procedure is usually carried out under ideal laboratory conditions and using standardized sample materials provided by the manufacturer. In this study, our first particular focus is the feasibility in transferring routine analysis methods from a second-generation sequencer, such as the Illumina MiSeq, to a fourth-generation system, such as the MinION of Oxford Nanopore Technology. The second aim is to evaluate whether the metabarcoding method can be transferred between sequencing platforms without modification or whether platform-specific adjustments are required. Moreover, it is necessary to assess whether comparable results can be achieved and what implications this has in terms of acquisition costs, cost reduction, handling, and maintenance. We therefore selected one of our own established and publicly available methods for the determination of pistachio in processed foods [15]. Thirdly, in addition, the quantitation possibilities on the MinION will be considered by using both MinION and Flongle flow cells. The method developed in this way is intended to sequence more samples in less time through easier handling, enable faster evaluation, be more cost-effective, and at the same time ensure accuracy comparable to existing approaches.

## 2. Materials and Methods

### 2.1. Samples and Sample Preparation

Reference samples include the following: *Pistacia vera*, *Juglans regia*, *Prunus dulcis*, *Helianthus annuus*, *Pisum sativum*, *Corylus* spp., and *Cucurbita* spp. To ensure the correct determination of the individual genus, these reference samples were collected from the Institute of Plant Sciences and Microbiology, Hamburg, University of Hamburg. These species are particularly suspected of being used to adulterate pistachios, as described above. In addition, retail samples of Pistacia spp., *Juglans* spp., *Prunus* spp., *Helianthus* spp., *Pisum* spp., *Corylus* spp., and *Cucurbita* spp. were used to prepare the control mixture for quantitation. The samples were ground and processed to bakery products. The nut layer was then separated and used for DNA extraction. For bakery products, with a focus on pistachio, a standard range of 0.1–100% has been produced. Therefore, the desired pistachio content was weighed, and a mixture of the remaining 6 genera was used as the sample matrix. In addition, commercial baklava samples, pistachio cremes/toppings, and pistachio protein/cereal bars were collected from stores in Hamburg between December 2024 and January 2025. At least five of each sample category. For all samples included, a set of technical and biological replicas were produced or purchased.

### 2.2. DNA Extraction and Amplicon PCR

**Figure 1:**
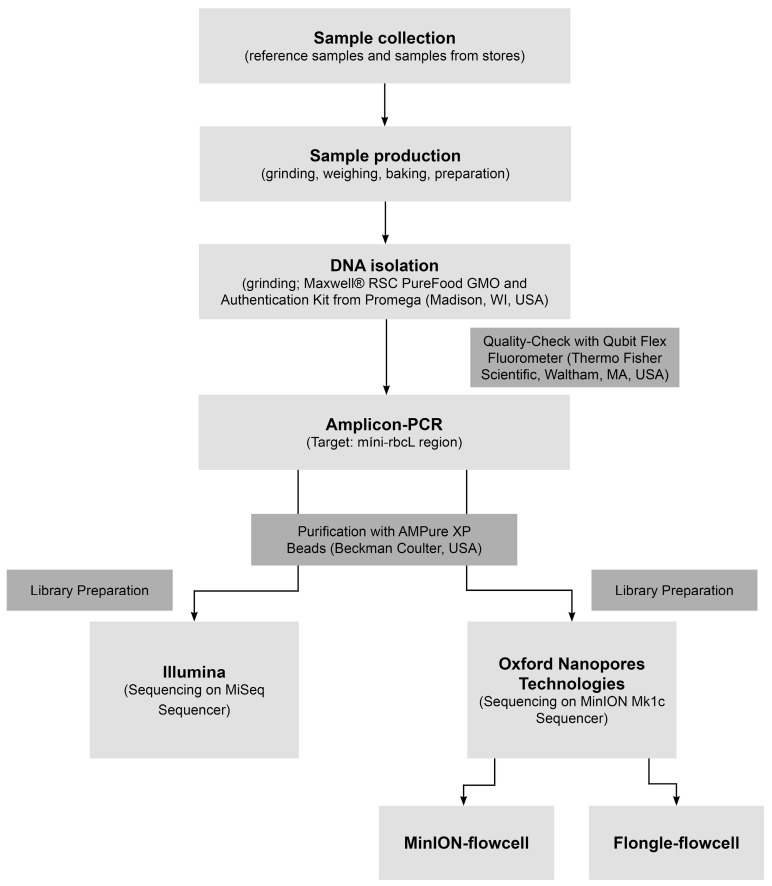
Experimental workflow without the bioinformatic analyses.

The experimental workflow without the bioinformatic analyses is shown in Figure 1.

After the collection/production, the samples were homogenized in different ways. The reference samples were crushed in a mortar and used for DNA extraction. The homogenization of commercial pistachio products has been classified into three categories (Table 1).

For homogenization the in-house established IKA tube mill control (IKA-Werke GmbH and Co. KG, Staufen, Germany) was used. Therefore approximately 5 g of the product was milled, depending on consistency with the addition of nuclease-free water.

The DNA extraction was performed using the Maxwell^®^ RSC PureFood GMO and Authentication Kit from Promega (Madison, WI, USA) following the manufacturer’s instructions. Approximately 10 mg of the homogenate was mixed with 1 mL of CTAB buffer, 40 µL of Proteinase K, and 20 µL of RNase in a 1.5 mL Eppendorf tube. The mixture was incubated for 90 min at 65 °C with shaking. After incubation, the mixture was centrifuged for 3 min at 14,000× *g* (model 5430 R, Eppendorf AG, Hamburg, Germany). Next, 300 µL of the supernatant and 300 µL of lysis buffer were added to the first well of the RSC cartridges, and the “PureFood GMO and Authentication Kit” program was started. The DNA isolates were stored at −20 °C.

For the next steps, the DNA concentrations of the extracts were determined with a Qubit Flex Fluorometer (Thermo Fisher Scientific, Waltham, MA, USA) by using the dsDNA HR Assay Kit (Thermo Fisher Scientific, Waltham, MA, USA).

For the amplicon PCR, all DNA extracts were adjusted to a concentration of 5 ng/μL. Respectively, 2.5 µL of each were used in the PCR. The reaction mix consisted of 12.5 μL of 2× HotStarTaq Master Mix Kit (Qiagen, Hilden, Germany), 1.75 µL of primers (forward and reverse, each at 2 14 μM), 3 μL of magnesium chloride solution (25 mM), and 3 μL of nuclease-free water.

The general mini-rbcL region primer set (including Illumina adapters) that was used was as follows (primer set in italics):

Forward (F52): TCGTCGGCAGCGTCAGATGTGTATAAGAGACAGGTTGGATTCAAAGCTGGTGTTA
Reverse (R193): GTCTCGTGGGCTCGGAGATGTGTATAAGAGACAGCVGTCCAMACAGTWGTCCATGT

The modified PCR program from Dobrovolny et al. [26] was applied and performed in a Touch Thermal Cycler (Biorad, Hercules, CA, USA) [26]. The amplicons were purified with AMPure XP Beads (Beckman Coulter, Brea, CA, USA) according to the manufacturer’s instructions, which involve two ethanol wash steps. A mixture of 20 µL of amplicon and 90 µL of beads was prepared. The purified amplicons were subsequently eluted in 53 µL of 10 mM Tris-HCL, pH 8.5.

### 2.3. Library Preparation ONT Seq

The library preparation was performed with the native barcoding kit 24 V14 (SQK-NBD114.24) and the rapid sequencing kit 24 V9 (SQK-RBK-110.96) from Oxford Nanopore Technologies (Oxford Nanopore Technologies, Oxford, UK) following the manufacturer’s instructions. Sequencing was performed on the MinION Mk1C platform using MinION and GridION flow cells R 10.4.1 (FLO-MIN114, respectively, Spot-ON flow cell R9 (FLO-MIN106D) and Flongle flow cells R 10.4.1 (FLO-FLG114) from Oxford Nanopore Technologies.

The start concentration of the amplicons for the native barcoding kit 24 V14 (SQK-NBD114.24) was 200 fmol in 11.5 µL nuclease-free water. A mixture of 1.75 µL Ultra II End-prep Reaction Buffer (New England Biolabs, Ipswich, MA, USA) together with 0.75 µL Ultra II End-prep Enzyme Mix (New England Biolabs) and 1 µL DNA control sample (DCS) was added. The reaction mixture was incubated first at 20 °C for 5 min, then at 65 °C for 5 min. This was followed by a further purification step using 15 µL AMPure XP Beads (Beckmann Coulter, Brea, CA, USA). The purified samples were eluted in 10 µL of nuclease-free water and 7.5 µL were used for the next step. For each sample 2.5 µL of an individual Native Barcode and 10 µL Blunt/TA Ligase Master Mix (New England Biolabs) were added. After 20 min incubation time 2 µL of EDTA (0.25 M) was added and the samples were pooled together. For each sample 9 µL AMPure XP Beads (Beckmann Coulter) mixed with the pool and another purification step was performed. In addition to the previous purification steps, the last step contains a 10 min incubation step at 37 °C. The final eluate was in 30 µL nuclease-free water. At the last library preparation step the pool was mixed with 5 µL Native Adapters, 5 µL Quick T4 DNA Ligase (New England Biolabs), and 10 µL NEBNext Quick Ligation Reaction Buffer (New England Biolabs). After a 20 min incubation time a last purification step with 20 µL AMPure XP Beads (Beckmann Coulter) was performed. This time, however, the wash steps were performed with 125 µL short fragment buffer (SFB) instead of ethanol. The final library was eluted in 15 µL elution buffer.

After flushing the respective flow cells, 10 fmol for Flongle flow cells R 10.4.1 (FLO-FLG114) and 45 fmol for MinION and GridION flow cells R 10.4.1 (FLO-MIN114) were loaded.

The start concentration of the amplicons for the rapid sequencing kit 24 V9 (SQK-RBK-110.96) was also 200 ng in 10 µL nuclease-free water. Each sample was mixed with 1 µL individual barcode. After incubating for 2 min at 37 °C and 2 min at 80 °C the samples were cooled down and pooled together. The same volume of AMPure XP Beads were added and the libraries were washed the same described above. The purified libraries were eluted in 15 µL elution buffer. Maximum 800 ng in 11 µL were mixed with 0.5 µL rapid adapter and 1.16 µL adapter buffer. After 5 min incubation the Spot-ON flow cell R9 (FLO-MIN106D) was flushed and the libraries were loaded. Biological and technical replicates were included in the study. To ensure robust validation, technical replicates were analyzed not only within a single sequencing run but also across two or more independent sequencing runs. This also applies to sequencing with Illumina.

### 2.4. Library Preparation and NGS

The library was prepared following the “16S Metagenomics Sequencing Library Preparation” protocol from Illumina (Illumina, San Diego, CA, USA), with some steps modified [26].

The reaction mixture for the index PCR contained 25 µL of 2× HotStarTaq Master Mix Kit (Qiagen), 10 µL IDT^®^ for Illumina^®^ DNA/RNA UD Indexes Set (Illumina) or Illumina^®^ DNA/RNA UD Index Set (Illumina), 10 µL nuclease-free water, and 5 µL of the previous produced amplicons. The index-PCR program was described in the protocol of Dobrovolny et al. [26]. A further purification step using 90 µL AMPure XP Beads (Beckmann Coulter) was performed. The final DNA library was dissolved in 28 µL of 10 mM Tris-HCL, pH 8.5. The concentration of the DNA was measured using the Qubit Flex Fluorometer (Thermo Fisher Scientific). The length of the final libraries was determined using the 4150 Tapestation System (Agilent, Santa Clara, CA, USA). For sequencing the final libraries were normalized to 4 nM, pooled together, and denaturated.

For sequencing, the concentration was adjusted to 8–10 pM and spiked with 15% of 12.5 pM PhiX control (Illumina). Paired-end sequencing was carried out on the MiSeq^®^ instrument using the 2 × 151 bp (300-cycle MiSeq^®^ Reagent Kit) Micro Kit v2 chemistry (Illumina).

### 2.5. NGS Data Analysis

#### 2.5.1. Data Analysis Oxford Nanopore Technologies

Oxford Nanopore Technologies provides a data analysis cloud service (EPI2ME) where different workflows for the evaluation of several subjects are available. For the metabarcoding approach the workflow “wf-alignment” (version 1.2.0) was selected. Therefore, the IT requirements include a minimum of 6 CPUs and 12 GB memory. For this, fastq files were used as input files. After setting up the path to a directory where the reference sequence in fasta format was located, the input reads are aligned by using Minimap2 [27]. A folder was generated, containing the whole chloroplast genomes of *Pistacia vera* (MN551174.1), *Prunus dulcis* (MT019559.1), *Juglans regia* (NC_028617.1), *Corylus avellane* (NC_031855.1), *Helianthus annuus* (NC_007977.1), *Cucurbita moschata* (OQ442842.1), and *Pisum sativum* (NC_014057.1) as reference sequences in fasta format. The depth of coverage over the whole reference sequence is determined by Mosdepth using 200 window per reference sequence [28].

#### 2.5.2. Data Analysis Illumina

For Illumina raw datasets, the pipeline APSCALE was used for evaluation [29]. For taxonomic assignment, OTUs were compared to the NCBI nt database [30] and a custom database using BLASTn 2.9.0 [31]. The OTU distribution was also used for quantitative evaluation [15].

**Figure 2:**
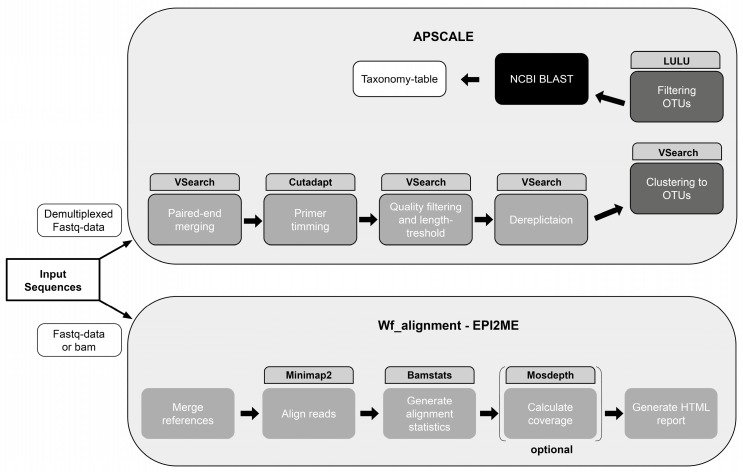
Graphical illustration of the pipeline APSCALE used for data output of Illumina sequencing and the wf_alignment workflow of EPI2ME used for Oxford Nanopore Technologies sequencing [29,32].

To illustrate the individual steps of the two bioinformatic pipelines (wf_alignment workflow of EPI2ME and APSCALE), they are shown graphically in Figure 2.

## 3. Results and Discussion

### 3.1. Results: Reference Samples and Standard Samples with ONT

#### 3.1.1. ONT wf_alignment_workflow

The reference samples of *Pistacia vera*, *Juglans regia*, *Prunus dulcis*, *Helianthus annuus*, *Pisum sativum*, *Corylus* spp., and *Cucurbita* spp. were sequenced with the MinION and GridION flow cell R 10.4.1 (FLO-MIN114), the Spot-ON flow cell R9 (FLO-MIN106D), and the Flongle flow cell R 10.4.1 (FLO-FLG114) on the MinION Mk1C platform. For the evaluation the wf_alignment workflow of EPI2ME with the custom database was used (Table 2).

For all samples, the assignment of the genus/species was correct. As only individual samples were sequenced, all content values should be at least 90%. This was not achieved with the Spot-ON flow cell R9 (FLO-MIN106D) in combination with the rapid sequencing kit 24 V9 (SQK-RBK-110.96). Therefore, it was decided not to pursue this kit/flow cell in further analysis. For all other rehearsals the 90% benchmark was achieved, except for *Prunus dulcis* with the Flongle flow cell and for *Cucurbita* spp. with both flow cells. For *Cucurbita* spp. the initial DNA isolates showed lower quality compared to the others, which may have contributed to the less accurate determination. For *Prunus dulcis* there was already in the previous trial a higher deviation [15]. However, this result is not shown for the MinION flow cell. In general, the results of the Flongle flow cell and the MinION flow cell did not differ significantly from each other for the reference samples. Even if the MinION flow cell produced much more data per sample (on average 344 Mb bases per sample compared to 21 Mb bases per sample), the quality of the bases/reads were adequate enough for both analyses. The values of the raw data are shown in Table 3. Despite the higher data volume, the percentage of passed reads was very similar (>84%) and the number of detected species remained comparable.

The custom database *hucustomdb_nuts_2* was used based on the experience that by using big databases, like the NCBI Refseq database, accurate classification at the species level with ONT is generally problematic because of the large misclassification or unclassified read numbers [1,15]. Moreover, the NCBI database is not curated for all subcategories, such as barcoding regions, and may contain duplicate copies of the same region, which can introduce bias in taxonomic assignment by overestimating certain regions due to their high abundance [8]. In addition, smaller reference databases can significantly improve the performance of ONT analysis by reducing computational load and memory requirements, enabling faster indexing and mapping [18]. This makes the evaluation process faster, which can lead to cost reductions. Moreover, focused databases tailored to the target organisms or genomic regions enhance specificity and signal-to-noise ratio [33]. Since this study was designed to replicate an established method while incorporating more efficient procedures in terms of speed and cost-effectiveness, the custom database approach was retained because it offers these advantages. In food authenticity, metabarcoding analyses represent a strong tool based on the provided robust results during species identification. For more details and since this is not the scope of this study, please check our previous published work for a comprehensive overview and comparison of metabarcoding analyses and NGS methodologies in distinguishment to other DNA-based methods [15].

**Figure 3:**
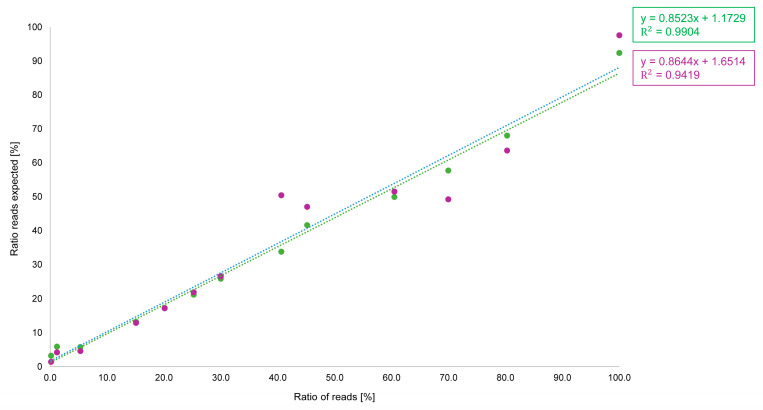
Ratio of reads sequenced with the Flongle flow cell (marked in purple) and MinION flow cell (marked in green) of produced bakery products with pistachio content from 0.1 to 100%.

For bakery products, with a focus on pistachio, a standard range of 0.1–100% has been produced. The bakery products (baklava) were sequenced in triplicate on the Flongle flow cell and the MinION flow cell, as well as in different sequencing runs. Figure 3 shows the results of the ratio of reads compared to the expected ratio of reads.

Both sequencing approaches show a linear progression. However, the results of the MinION flow cell are significantly better, as can be seen from the regression measurement. Therefore, the MinION flow cell not only provides larger amounts of data, but also more accurate quantitative determinations. Therefore, the MinION flow cell was used for the direct comparison with the MiSeq data of Illumina. For our general thesis, the transfer of a routine analysis for the identification of pistachio in bakery products from a second-generation platform to a fourth-generation platform, sequencing with a Flongle flow cell is sufficient, as it provides adequate data and is significantly less expensive than the MinION flow cell.

#### 3.1.2. Comparison of Illumina and Oxford Nanopore Technologies

**Figure 4:**
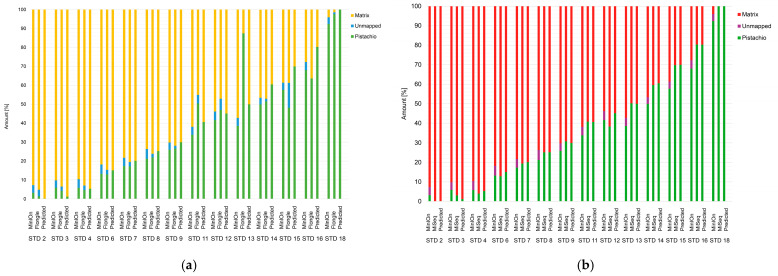
Comparison of ratio of reads in dependance of chosen sequencing platform. Ratio of reads of produced bakery products with pistachio content from 0.1 to 100% sequenced with Flongle flow cell and the MinION flow cell (**a**) in comparison to ratio of reads sequenced with Oxford Nanopore Technologies (MinION flow cell) and the Miseq from Illumina (**b**).

To demonstrate the possibility of transferring routine analysis methods from a second-generation sequencer, such as the MiSeq from Illumina, to a fourth-generation system, such as the MinION from Oxford Nanopore Technology, the exact same samples were selected from the validation of the existing method [15]. In more detail the samples were sequenced on the Flongle flow cell and the MinION flow cell from ONT and on the V2 Micro flow cell from Illumina. The results are illustrated in Figure 4. The two sequencing platforms were evaluated using two different pipelines (APSCALE for Illumina and EPI2ME (wf_alignment) for ONT), as the output data differed so much in quality and format (FASTQ and FAST5) that the pipelines had to be adapted to the respective method [8].

The proportion of unassigned reads in the MiSeq sequencing evaluation was minimal, so they are not visible in the diagram. Figure 4 shows that the MinION flow cell data and MiSeq data are more closely aligned compared to the Flongle flow cell data and MinION flow cell data, which may be attributed to the two outliers present in the Flongle dataset (STD11 and STD13). Since the same samples were measured multiple times on different instruments and flow cells, it can be assumed that these are outliers from the Flongle flow cell and not errors in sample preparation.

**Figure 5:**
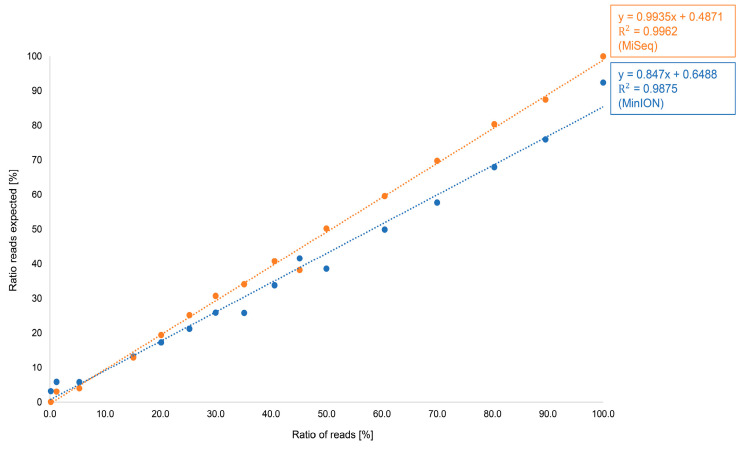
Ratio of reads of produced bakery products with pistachio content from 0.1 to 100% sequenced with the MinION flow cell on Mk1C sequencer from Oxford Nanopore Technologies (marked in blue) and the Micro flow cell on MiSeq sequencer from Illumina (marked in orange).

Figure 5 shows the results from the Oxford Nanopore Technologies platform (Mk1C; MinION flow cell) and the Illumina platform (MiSeq; V2 Micro flow cell) in more detail. The regression analysis demonstrates that the MiSeq data are closer to the expected values than the ONT data. ONT data typically have a higher error rate than Illumina, even though the company has improved these values in the past and is still working on achieving better values [1,8]. With large genomes, this can lead to particular problems and require higher coverage. However, the amplicons analyzed in this study are comparatively short (approximately 300 bp) and thus yield a high read depth, which helps to mitigate errors in the base sequence. As we were able to show with our results, the accuracy of the ONT data is sufficient for the method used in this case.

This statement is further supported by measurements on commercial baklava samples. The exact same samples from the previous analysis on Illumina MiSeq were measured on an ONT MinION flow cell (Table 4). Slight deviations in the measurement results are also evident in this case, but a semi-quantitative statement about the general composition of the sample is possible.

One explanation for the deviation may be the Q-score. The Q-score quantifies the error probability on a logarithmic scale. It is typically specified with Q30 (99.9% accuracy) for Illumina and Q7–Q10 for Oxford Nanopore Technologies, with the current new “super accuracy” basecalling models even reaching Q15–Q20 (90–99% accuracy) [10,34,35]. Although the measurements were predominantly performed with the new chemicals for Q20 scores, they only achieved an average >Q8–9-score within the majority of reads/bases. With Illumina sequencing, always at least 90% of the data achieves a Q30 value. However, the Q-scores of the two sequencing technologies cannot be directly compared, as Illumina’s Q-scores are very directly related to base accuracy, while ONT’s Q-scores originate from base caller models and therefore reflect only model uncertainty and not just measurement noise. Therefore, Q-scores are very high and stable at Illumina, usually distributed evenly across the reads, and tend to be lower at ONT, where they can vary greatly within a read. This depends on pore behavior, DNA movement, and signal noise [36].

As shown in Figure 2, the evaluation with the APSCALE pipeline, which is only one of many applicable pipelines, provides a more detailed assessment than the EPI2ME pipeline. EPI2ME pipelines frequently include predefined default parameters—such as quality filters, adapter trimming, and settings of alignment parameters—while the user control is generally limited to the initial configuration of read-quality parameters. Moreover, the format of the final output containing the taxonomic assignment results is not compatible with the tools commonly used for downstream analyses [8]. Although open-source pipelines for processing ONT data are actively developed, their adoption remains relatively limited. In this case, only one workflow (wf_alignment) was suitable for evaluating the samples. Illumina also offers its own pipelines, which are certainly just as opaque as those provided by ONT, but working with Illumina sequences is already so well established that a large number of open-source pipelines are available. The advantages of EPI2ME pipelines are that they do not require any prior knowledge of bioinformatics for installation and have a very fast analysis time for the short barcoding regions evaluated here (approx. 10 min for 50 samples).

In addition, ONT sequencing requires a substantially lower initial DNA concentration for library preparation compared to Illumina sequencing platforms. This feature can be advantageous, particularly under challenging PCR conditions (e.g., the presence of inhibitory matrix components) where the starting concentration of the template material is low. Nevertheless, the amplified products must have sufficient integrity and quality to be suitable for sequencing. Excessive degradation of the samples can hinder PCR amplification or result in DNA fragments that are too short for effective sequencing [37]. When comparing the two sequencing methods used here with the respective kits (Illumina: Micro flow cell; ONT: MinION flow cell), the methods are similarly expensive at full capacity. Both kits are designed for a maximum of 96 samples (depending on the indices), which is the limiting factor here. However, for smaller sample quantities, the Flongle flow cell is a very good, cost-effective alternative. Both platforms are more cost-effective than classic PCR approaches as they are applicable for multiplex approaches [20]. Library preparation steps are generally a little faster with Illumina library preparation, as there are fewer and simpler steps.

The acquisition costs and environmental requirements are also lower at ONT. The flow cells of ONT can be recycled but if not, no extra wash step or other maintenance steps are required. At Illumina, the MiSeq instrument must be washed regularly but in newer systems this step has been omitted. Illumina sequencing technology has been established for a comparatively longer period, which has contributed to the company’s strong market presence and reputation. The platform offers a range of professional customer service and a substantial body of the scientific literature is based on data generated using Illumina systems.

### 3.2. Results: Pistachio Products

The results of the pistachio products are shown in Table 5. Sequencing was performed on the Mk1C platform with the MinION flow cell (ONT) and on the MiSeq platform with the Micro Kit v2 chemistry (Illumina).

**Table 5:** Homogenization categories and results of selected pistachio products.

Sample ID	Categories of Homogenization	Pistachio Content Declared [%]	Amount Detected [%] MinION	Amount Detected [%] MiSeq
PP1	1	17	65.2	74.3
PP2	2	16	83.9	92.3
PP3	2	25	98.2	100
PP4	2	30	98.3	99.8
PP5	2	35	96.5	100
PP6	2	25	97.4	100
PP7	2	100	97.3	100
PP8	1	17	65.7	72.2
PP9	1	12	76.1	82.1
PP10	1	15	58.4	67.3
PP11	1	15	44.4	52.8

The pistachio content stated on the products does not correspond to the proven pistachio quantity. This was higher in every sample, even when taking into account a high range of deviation in the information provided. This scheme is evident on both sequencing platforms, which indicates a significant bias in the PCR process. The mini-rbcL-barcoding region was specifically selected based on pistachio and other nut species and shows a strong match with these particular plant DNA sequences. The primers used are not compatible with DNA from other ingredients, such as animal DNA found in milk. Consequently, only plant DNA is amplified and subsequently sequenced. To address these biases, it is common practice to combine multiple DNA barcoding regions, such as 16S region for animal DNA, to enable a more comprehensive analysis [38]. Furthermore, despite the use of supposedly universal primers, primer–template mismatches can occur due to gene variability, even in conserved regions, which can distort amplification and potentially impact identification, especially in mixed samples containing multiple genera [1]. The results support the thesis that DNA-based methods should always be validated on the respective matrices. A second attempt was made to sequence the entire DNA directly, without prior PCR amplification. However, the results were suboptimal due to a high proportion of unmapped reads, which compromised the accuracy of the quantitative analysis.

However, since the aim of this study was to investigate whether the previous “one barcode approach” of the previous baklava method could be adopted, the multi-barcode approach was not implemented [15]. In the previous baklava analysis method, only the nut layer was examined, which contained minimal DNA in addition to plant DNA. The other ingredients consisted exclusively of sugar and oil. As a result, the bias is less significant, permitting a semi-quantitative analysis. For products other than Baklava (Table 3), the mini-rbcL region can only be used for qualitative analysis.

## 4. Conclusions

In summary, the existing routine analysis method for detecting pistachio fraud could be transferred from a second-generation sequencer, Illumina MiSeq, to a fourth-generation system, MinION of Oxford Nanopore Technology. Since the DNA fragments for the metabarcoding method are very short, the quality of ONT sequencing is perfectly adequate. Both flow cells (MinION and Flongle flow cell) achieve almost the same quality as Illumina sequencing with the Miseq. While Illumina sequencing offers advantages such as higher accuracy, user-friendly operation, and more robust data analysis options, Oxford Nanopore Technologies provides competitive benefits in other areas, including sequencing speed, low maintenance requirements, and reduced costs. The Flongle flow cell in particular is an extremely cost-effective alternative, especially for applications involving low sample throughput. Products with a complex matrix, like cereal bars or cremes, could not be validated. However, the nut layer from the baklava matrix was successfully confirmed in quality and semi-quantitative analyses.

## Figures and Tables

**Table 1:** Categories of homogenization and description.

Category	Description	Homogenization	Example
1	The pistachio content refers to the entire product, although pistachios are only present in certain parts	The whole product has been homogenized and nuclease-free water has been added (if necessary)	Protein bar, cereal bar
2	The term “pistachio” refers to the whole product and the product is homogenous	Homogenization by stirring	Creams toppings
3	The pistachio content refers only to parts of the product	The portion of the product has been isolated and homogenized (with nuclease-free water if necessary)	Bakery products

**Table 2:** Results of the reference samples on the MinION Mk1C platform with MinION and GridION flow cell R 10.4.1 (FLO-MIN114) and the Flongle flow cells R 10.4.1.

Reference Material	Flongle Flow Cell (FLO-FLG114)		MinION Flow Cell (FLO-MIN114)		Spot-ON Flow Cell (FLO-MIN106D)	
	Species Detected	Amount [%]	Species Detected	Amount [%]	Species Detected	Amount [%]
*Pistacia vera*	*Pistacia vera*	93.6	*Pistacia vera*	94.1	*Pistacia vera*	83.3
*Juglans regia*	*Juglans regia*	97.5	*Juglans regia*	97.2	*Juglans regia*	88.2
*Prunus dulcis*	*Prunus dulcis*	78.4	*Prunus dulcis*	97.0	*Prunus dulcis*	79.4
*Helianthus annuus*	*Helianthus annuus*	97.2	*Helianthus annuus*	95.6	*Helianthus annuus*	88.2
*Pisum sativum*	*Pisum sativum*	96.1	*Pisum sativum*	94.6	*Pisum sativum*	80.6
*Corylus* spp.	*Corylus avellana*	95.9	*Corylus avellana*	97.0	*Corylus avellana*	81.1
*Cucurbita* spp.	*Cucurbita moschata*	74.0	*Cucurbita moschata*	79.2	*Cucurbita moschata*	58.9

**Table 3:** Values of the raw data without evaluation of the Flongle flow cell and the MinION flow cell sequencing.

	Flongle Flow Cell					MinION Flow Cell				
	Total Bases [Mb]	Passed Bases [%]	Total Reads [k]	Passed Reads [%]	Amount [%]	Total Bases [Mb]	Passed Bases [%]	Total Reads [k]	Passed Reads [%]	Amount [%]
*Pistacia vera*	25.1	84.4	69.7	85.1	93.6	410.1	86.7	1071.7	89.2	94.1
*Juglans regia*	9.9	86.2	28.4	86.5	97.5	447.4	86.8	1163.1	90.8	97.2
*Prunus dulcis*	4.4	85.2	12.9	86.1	78.4	483.8	86.3	1256.1	89.1	97.0
*Helianthus annuus*	46.8	84.8	129.2	85.1	97.2	276.8	87.4	744.5	89.6	95.6
*Pisum sativum*	26.6	85.4	74.7	86.1	96.1	380.9	88.1	980.3	91.3	94.6
*Corylus* spp.	30.6	85.3	84.8	85.8	95.9	346.7	85.8	899.1	89.1	97.0
*Cucurbita* spp.	5.3	85.8	12.4	84.8	74.0	62.4	80.5	136.1	87.1	79.2

**Table 4:** Results of the commercial baklava samples sequenced with MinION flow cell on ONT and Micro flow cell on Illumina MiSeq.

Sample ID	Genus Declared in the Nut Layer [%]	Amount Detected [%] MinION	Amount Detected [%] MiSeq ^1^
B1	*Pistacia* spp.	54.5	61.0
B2	*Pistacia* spp.	59.2	64.6.
B3	*Pistacia* spp.	21.2	31.7
B4	*Pistacia* spp.	62.4	72.5
B5	*Pistacia* spp.	65.2	70.4
B6	*Pistacia* spp.	47.6	54.4
B7	*Pistacia* spp.	2.2	0.5
B8	*Pistacia* spp.	2.8	5.2
B9	*Pistacia* spp.	15.6	13.2
B10	*Pistacia* spp.	83.9	97.8
B11	*Pistacia* spp.	85.0	99.9
B12	*Pistacia* spp.	87.9	98.8

^1^ Published results from Rammouz et al., 2025 [15].

## Data Availability

The original contributions presented in the study are included in the article, further inquiries can be directed to the corresponding author.
